# Quantitative Proteomic Analysis Uncovers the Mediation of Endoplasmic Reticulum Stress-Induced Autophagy in DHAV-1-Infected DEF Cells

**DOI:** 10.3390/ijms20246160

**Published:** 2019-12-06

**Authors:** Jingjing Lan, Ruihua Zhang, Honglei Yu, Jingyu Wang, Wenxiang Xue, Junhao Chen, Shaoli Lin, Yu Wang, Zhijing Xie, Shijin Jiang

**Affiliations:** 1 College of Veterinary Medicine, Shandong Agricultural University, Taian 271000, China; jjlan1024@163.com (J.L.); ruirui041127@126.com (R.Z.); 17863805800@163.com (H.Y.); jywang676@163.com (J.W.); 15094796173@163.com (W.X.); junhao-chenyy@163.com (J.C.); xiezhj@sdau.edu.cn (Z.X.); 2 Shandong Provincial Key Laboratory of Animal Biotechnology and Disease Control and Prevention, Taian 271000, China; 3 College of Public Health and Management, Weifang Medical University, Weifang 261042, China; 4 Molecular Virology Laboratory, VA-MD College of Veterinary Medicine and Maryland Pathogen Research Institute, University of Maryland, College Park, MD 20742, USA; lsl1990@umd.edu; 5 Department of Basic Medical Sciences, Taishan Medical College, Taian 271000, China; wangyu1974-1@163.com

**Keywords:** duck hepatitis A virus type 1, quantitative proteomics, differentially expressed proteins, host–pathogen interaction, ER stress-induced autophagy

## Abstract

Autophagy is a tightly regulated catabolic process and is activated in cells in response to stress signals. Despite extensive study, the interplay between duck hepatitis A virus type 1 (DHAV-1) and the autophagy of host cells is not clear. In this study, we applied proteomics analysis to investigate the interaction mechanism between DHAV-1 and duck embryo fibroblast (DEF) cells. In total, 507 differentially expressed proteins (DEPs) were identified, with 171 upregulated proteins and 336 downregulated proteins. The protein expression level of heat shock proteins (Hsps) and their response to stimulus proteins and zinc finger proteins (ZFPs) were significantly increased while the same aspects of ribosome proteins declined. Bioinformatics analysis indicated that DEPs were mainly involved in the “response to stimulus”, the “defense response to virus”, and the “phagosome pathway”. Furthermore, Western blot results showed that the conversion of microtubule-associated protein 1 light chain 3-I (LC3-I) to the lipidation form of LC3-II increased, and the conversion rate decreased when DEF cells were processed with 4-phenylbutyrate (4-PBA). These findings indicated that DHAV-1 infection could cause endoplasmic reticulum (ER) stress-induced autophagy in DEF cells, and that ER stress was an important regulatory factor in the activation of autophagy. Our data provide a new clue regarding the host cell response to DHAV-1 and identify proteins involved in the DHAV-1 infection process or the ER stress-induced autophagy process.

## 1. Introduction

Duck hepatitis A virus (DHAV) is a member of the genus *Avihepatovirus* in the Picornaviridae family. DHAV is divided into three serotypes, namely, the world-wide traditional serotype called DHAV-1 [1,2], a serotype isolated in Taiwan called DHAV-2 [3], and a serotype isolated in South Korea and China called DHAV-3 [4,5]; no antigenic relationships have been found among them [3]. As a fatal rapidly spreading disease, DHAV-1 infection is characterized by liver petechiae and hepatitis in young ducklings and egg drop in laying duck flocks [6,7,8,9]. In order to control DHAV-1 infection, researchers have made great efforts to study the interactive mechanism between DHAV-1 and host cells [10,11,12].

Autophagy is a traditional mechanism that degrades protein and waste in cells. It has been reported previously that the endoplasmic reticulum (ER), as an integral and elaborate organelle for folding and modifying secretory proteins, can induce autophagy if it is damaged [13]. Viral infection can lead to disorder of the intracellular environment, such as the accumulation of misfolded protein or unfolded protein in ER or Ca^2+^ balance, so cells launch the ER stress and autophagy reaction against the infection. ER autophagy is a selective autophagy process with a key role in regulating the unfolded protein response (UPR), which is responsible for maintaining cell homeostasis [14]. Researchers have affirmed that ER stress and autophagy participate in numerous cell processes during virus infection, such as cell death, the immune response, and viral replication [15,16,17]. Moreover, Toll-like receptor and type I interferon production are triggered by autophagosome fusion with the lysosomal pathway [18]. Therefore, it is not surprising that viruses have evolved some evasion mechanisms to achieve infection. Recent studies have shown that some viruses can inhibit or evade autophagy, whereas some viruses can not only induce autophagy but even take advantage of it to promote virus replication [19,20,21]. Although DHAV-1 has been reported to induce apoptosis and the immune response [22,23], it is still necessary to find more sufficient evidence to elucidate the phenomenon in ER stress-induced autophagy, especially because there are currently no reports on this process.

Proteomic methods are a highly specific, effective, and universal technique, which do not require multi-step sample preparation [24]. Compared to RNA-seq, proteomic techniques can accurately reflect the abundances of downstream proteins, thus strategies focusing on protein quantification or/and post-translational modification have been widely applied in this area [25,26]. To date, several studies have focused on the interaction of viruses and host cells based on the proteomic method [27,28,29,30]. In this study, we focused on proteome changes of host proteins that were possibly involved in ER stress-induced autophagy in duck embryo fibroblast (DEF) cells, which are a natural primary target for DHAV-1. The quantification results, followed by gene ontology (GO) and Kyoto Encyclopedia of Genes and Genomes (KEGG) pathway analysis, showed that differentially expressed proteins (DEPs) were mainly involved in cellular processes, cell stimulation, the immune response, lysosomes, phagosomes, and others. Transmission electron microscopy (TEM) analysis confirmed that double-membraned autophagy-like vesicles were generated in DHAV-1-infected DEF cells. Proteomics results and Western blot results indicated that DHAV-1 was involved in the process of ER stress-induced autophagy. Overall, these findings enhance the understanding of the pathogenic mechanism at the cellular level during DHAV-1 infection.

## 2. Results

### 2.1. DHAV-1 Infection

**Figure 1:**
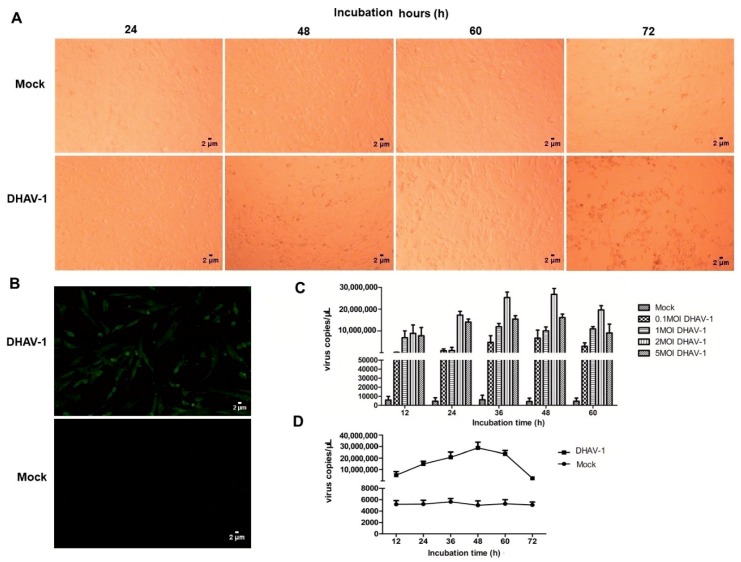
Characteristics of duck hepatitis A virus 1 (DHAV-1) infection on duck embryo fibroblast (DEF) cells. (**A**) Cell morphology of DEF cells with or without DHAV-1 infection were observed at 24, 48, 60, and 72 hpi. (**B**) DHAV-1 successfully infected DEF cells. (**C**) The mRNA copy numbers of DHAV-1 on DEF cells with different multiplicity of infection (MOI) at 12, 24, 36, 48, and 60 hpi. (**D**) The mRNA copy numbers of DHAV-1 on DEF cells with a MOI of 2 at 12, 24, 36, 48, 60, and 72 hpi.

After DHAV-1 infection, the cell morphologies were observed. The results showed that DEF cells shrunk at 48 h post-infection (hpi) and shedded at 60 hpi (Figure 1A). An indirect immunofluorescence assay (IFA) showed that DHAV-1 successfully infected DEF cells, observed by green fluorescence, while the non-infected DEF cells showed no fluorescence (Figure 1B). The qRT-PCR results showed that the optimal amount of DHAV-1 infection was a multiplicity of infection (MOI) of 2 (Figure 1C). The messenger RNA (mRNA) copies of DHAV-1 reached a maximum at 48 hpi, decreased at 60 hpi, and reached the lowest level at 72 hpi (Figure 1D). Therefore, DEF cells at 48 hpi were chosen to further investigate the changes in the host proteins during DHAV-1 infection.

### 2.2. Tandem Mass Tag (TMT)-Based Proteomic Analysis

**Figure 2:**
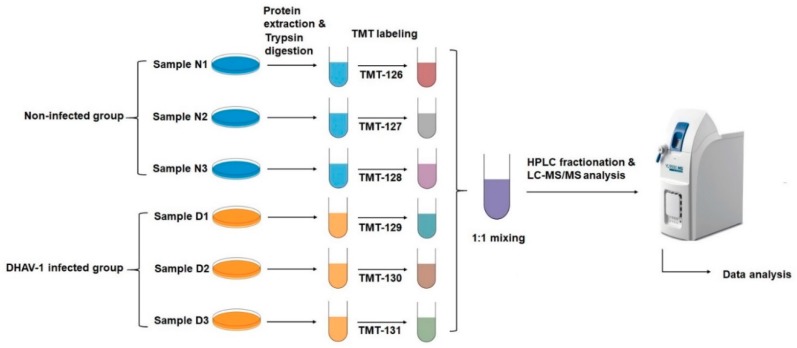
A schematic diagram of the experimental design flow. Proteins were extracted from DHAV-1-infected DEF cells and non-infected DEF cells. N: Non-infected DEF cells; D: DHAV-1-infected DEF cells. Experiments were performed as biological triplicates.

**Figure 3:**
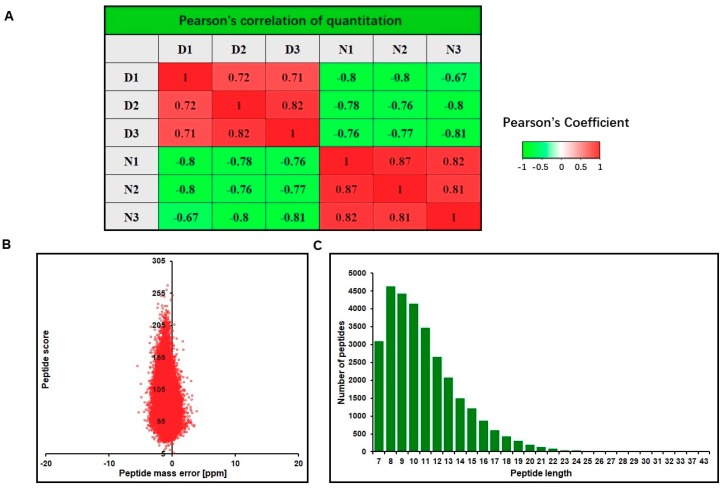
Quality control validation of mass spectrometry (MS) data. (**A**) Mass data of all identified peptides. It is a negative correlation or positive correlation when the Pearson coefficient is closer to -1 or 1, respectively. The green indicates the Pearson coefficient closer to -1, while the red indicates the Pearson coefficient closer to 1. (**B**) Average peptide mass error. (**C**) Length distribution of all identified peptides.

Based on TMT labeling-based proteomics technology, we aimed to investigate the global pattern of DEF cells either infected with DHAV-1 or not. Our experimental workflow is shown in Figure 2. In the identification, the pairwise Pearson’s correlation coefficients were greater than 0.7, suggesting sufficient reproducibility of the experiment (Figure 3A). From tandem mass spectrometry (LC-MS/MS) analysis, a total of 273,692 (47,151 matched) spectra were obtained. Of these spectra, 29,975 identified peptides (28,990 unique peptides) and 5250 identified proteins (4573 quantified proteins) were detected (Table 1, Table S1), and the average mass error of the peptides was <10 ppm, suggesting a high mass accuracy of the MS data (Figure 3B). In accordance with the properties of tryptic peptides, the length of the peptides was mainly distributed between 8 and 20 amino acid residues (Figure 3C), confirming that the sample preparation met the standard for further analysis. DEPs with significant change (*p*-value < 0.05) were selected, where the cut-off point was >1.3-fold change (*p*-value < 0.05) for upregulated proteins and <0.77-fold change (*p*-value < 0.05) for downregulated proteins. A total of 507 DEPs were identified, including 171 upregulated proteins and 336 downregulated proteins (Table S2).

### 2.3. Functional Classification and Subcellular Location Analysis of DEPs

**Figure 4:**
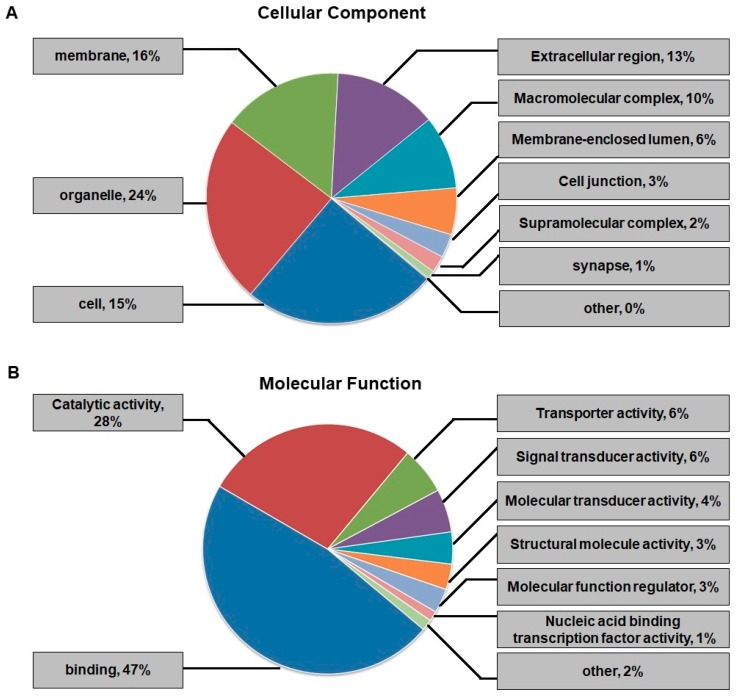

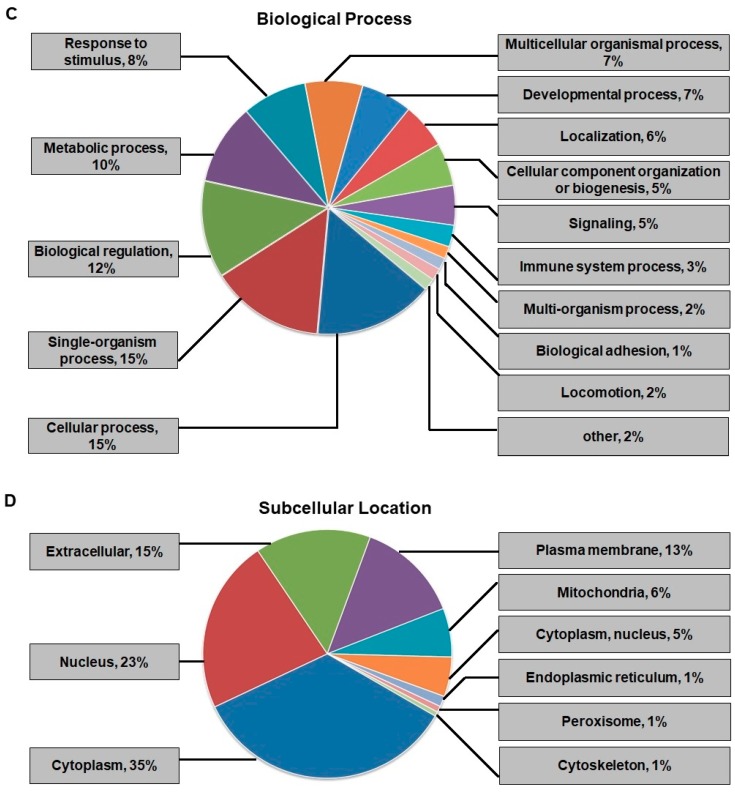
Gene ontology (GO) functional classification and subcellular functional annotation of differentially expressed proteins (DEPs). (**A**) GO annotation in terms of cellular components. (**B**) GO annotation in terms of molecular function. (**C**) GO annotation in terms of biological processes. (**D**) Subcellular locations of DEPs. GO: Gene ontology.

To characterize the functions and subcellular locations of DEPs between the DHAV-1-infected cells (D group) and the non-infected cells (N group), GO functional classification was performed and subcellular locations were analyzed (Figure 4, Tables S3 and S4). DEPs were divided into three different groups according to the cell component, molecular function, and biological process. The cell component results showed that the DEPs were mainly distributed in cells (25%), organelles (24%), the membrane (16%), and the extracellular region (13%) (Figure 4A). Regarding molecular function, the largest two GO categories were binding and catalytic activity, accounting for 47% (330 DEPs) and 28% (193 DEPs), respectively (Figure 4B). Biological process was the category we were most concerned with; DEPs were involved in cellular processes, single-organism processes, and biological regulation, accounting for 15%, 15%, and 12%, respectively (Figure 4C). These results demonstrated that the DEPs identified in the study participated in multiple cellular components, diverse molecular functions, and varied biological processes. Subcellular location predictions showed that the DEPs were mainly distributed in the cytoplasm (35%) and the nucleus (23%) (Figure 4D), highlighting that the two organelles play significant roles in DHAV-1 infection.

### 2.4. Enrichment Analysis of DEPs in GO, KEGG, and Protein Domain

**Figure 5:**
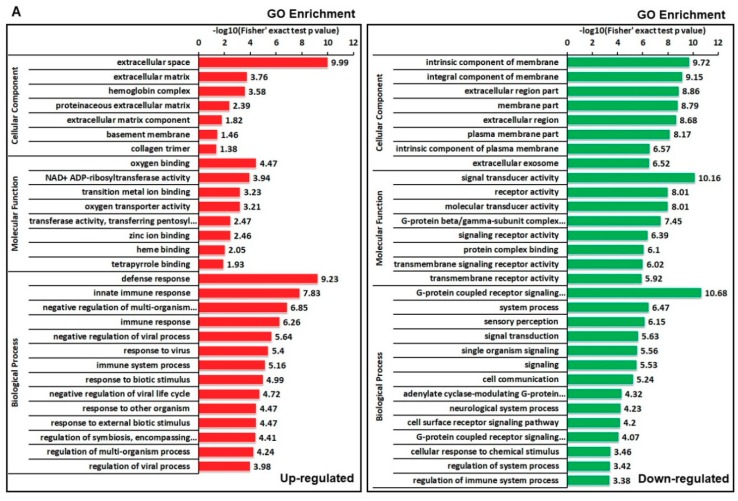

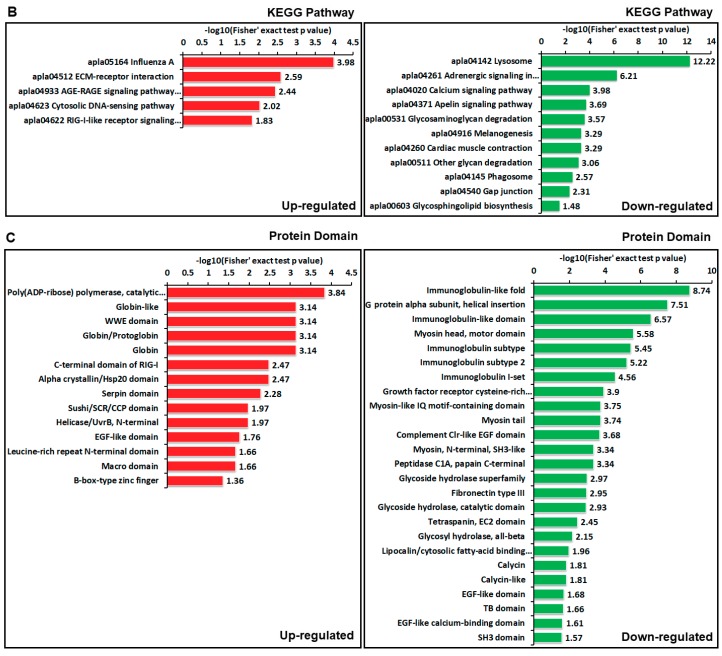
Enrichment clustering analyses of DEPs. (**A**) DEPs were classified by GO enrichment based on the following three categories: biological processes, cellular components, and molecular function. (**B**) DEPs were annotated based on the Kyoto Encyclopedia of Genes and Genomes (KEGG) pathway database. (**C**) DEPs were annotated based on the protein domain database.

To gain greater insight into the preferred functional enrichment of DEPs in response to DHAV-1 infection, three types of enrichment-based analyses were performed, namely, GO, the KEGG pathway, and protein domain analysis (Figure 5). We tested the dataset for enrichment in three GO categories, i.e., cellular components, molecular function, and biological processes (Figure 5A, Tables S5 and S6). Within the cellular component category, the proteins involved in the extracellular space were upregulated while intrinsic components of the membrane-related proteins were downregulated. Molecular function analysis showed that DEPs involved in oxygen binding and signal transduction were enriched in up- and downregulated protein clusters, respectively. Regarding biological processes, both up- and downregulated proteins were distributed in various metabolic processes. Notably, proteins related to the immune response, heat shock proteins (Hsps), zinc finger proteins (ZFPs), and proteins involved in the response to biotic stimuli were significantly enriched in upregulated proteins, whereas ribosomal proteins were decreased (Table 2). We speculate that DHAV-1 infection causes the accumulation of folded proteins and then activates ER stress in DEF cells.

The KEGG pathway analysis revealed that lysosomes and phagosomes were significantly enriched, except in the immune-related pathway (Figure 5B, Tables S7 and Table S8). Combined with the results showing sequestosome 1 (SQSTM1/p62) and death-associated protein kinase (DAPK) proteins were significantly enhanced, we speculate that DHAV-1 mediates the autophagy process. Domain enrichment analysis showed that the “poly (ADP-ribose) polymerase (PARP), catalytic domain”, and “Globin-like” were enriched in upregulated proteins, whereas the “immunoglobulin-like domain” and “G protein alpha subunit, helical insertion” were extremely abundant in downregulated proteins (Figure 5C, Tables S9 and S10).

### 2.5. Protein Interaction Network Analysis between DEPs and Ribosome Proteins

**Figure 6:**
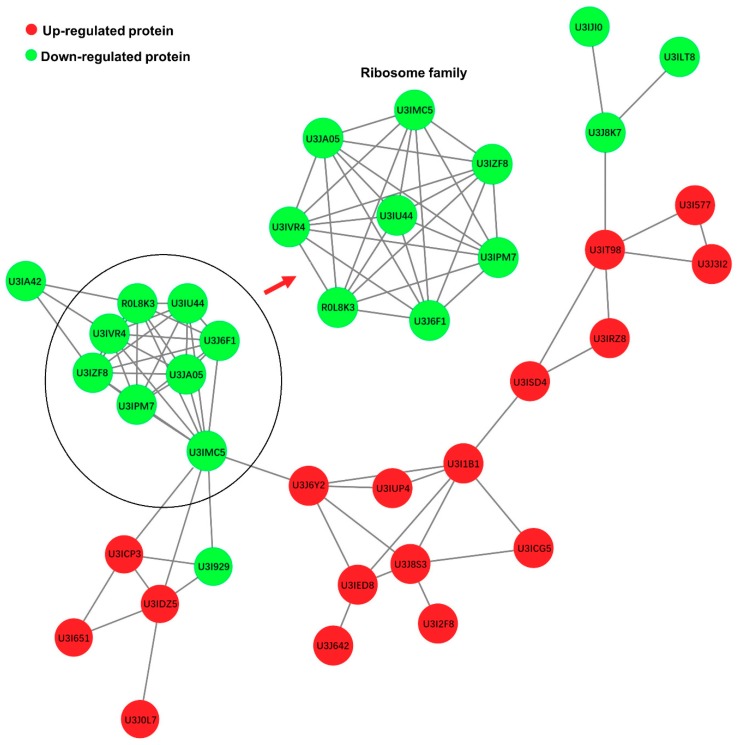
Interaction network between DEPs and ribosome proteins when comparing the D group to the N group. Red nodes represent upregulated DEPs while green nodes represent downregulated DEPs.

A network analysis of protein–protein interactions was performed to investigate the important interactions between DEPs (Table S11). The results revealed tight communications among ribosome proteins (green node), immune-related proteins (U3I1B1 (IFIT5, interferon-induced protein with tetratricopeptide repeats 5), U3J8S3 (*MX1*, myxovirus resistance gene 1), U3J642 (IFIH1, interferon-induced helicase), and U3J6Y2 (*ISG15*, interferon-stimulated genes 15)) and response to stimulus proteins (U3IED8 (RSAD2, Radical S-adenosyl methionine domain containing 2), U3IRZ8 (PARP9), U3J8K7 (Notch2), and U3IJI0 (ADAM17, ADAM metallopeptidase domain 17)), and highly connected clusters in ribosome proteins (Figure 6).

### 2.6. Validation of TMT/MS Data Using Alternative Methods

**Figure 7:**
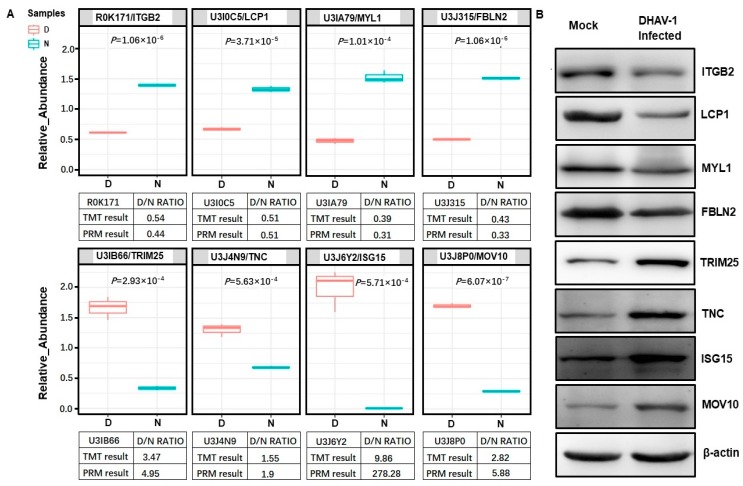
Verification of the DEPs by parallel reaction monitoring (PRM) and Western blot. (**A**) DEPs, including integrin beta 2 (ITGB2), lymphocyte cytosolic protein 1(LCP1), myosin light chain 1 (MYL1), fibulin 2 (FBLN2), tripartite motif containing 25 (TRIM25), tenascin C (TNC), interferon-stimulated genes 15 (ISG15), and Mov10 RISC complex RNA helicase (MOV10), in DHAV-1-infected and non-infected DEF cells were verified using PRM analysis. (**B**) Western blot analysis was used to reconfirm the expression level of the above-mentioned proteins. The expression trends of the DEPs that were identified in Western blot analysis matched those observed in the proteomics data.

To verify the TMT/MS data, we performed parallel reaction monitoring (PRM) analysis for eight selected DEPs (four upregulated proteins and four downregulated proteins). The relative abundance data are presented as a linear box-and whiskers plot and the expression of all identified DEPs was identical to the proteomic data (Figure 7A). The value of the D/N ratio between PRM and TMT/MS showed no noticeable differences in DEPs, except the ISG15 protein, which may be a reflection of the different detection methods. To further verify the TMT/MS data, a Western blot assay was performed and the results showed that integrin beta 2 (ITGB2), lymphocyte cytosolic protein 1 (LCP1), myosin light chain 1 (MYL1), and fibulin 2 (FBLN2) were downregulated in DHAV-1-infected cells, whereas tripartite motif containing 25 (TRIM25), tenascin C (TNC), ISG15, and Mov10 RISC complex RNA helicase (MOV10) were upregulated (Figure 7B). These findings were consistent with our TMT/MS data. Taken together, both the PRM and the Western blot results supported our TMT/MS data.

### 2.7. Induction of ER Stress-Induced Autophagy by DHAV-1 Infection

**Figure 8:**
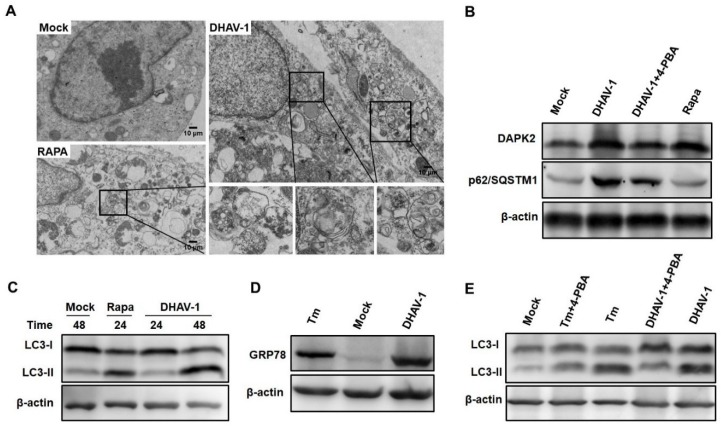
Mediation of ER stress-induced autophagy by DHAV-1. (**A**) The formation of double-membraned autophagy-like vesicles in DEF cells with mock infection, DHAV-1 infection, and rapamycin (RAPA) treatment was observed. (**B**) Expression levels of DAPK2 and p62/SQSTM1 were identified using Western blot. (**C**) Western blot detected the transformation from microtubule-associated protein 1 light chain 3-I (LC3I) to light chain 3-II (LC3II) caused by DHAV-1 infection in DEF cells. (**D**) Expression level analysis of the ER stress marker protein glucose-regulated protein 78 (GRP78) in DHAV-1-infected DEF cells. (**E**) Inhibiting ER stress could decrease the transformation from LC3I to LC3II caused by DHAV-1 infection.

To further verify the speculation whether DHAV-1 infection mediates ER stress-induced autophagy, we first observed the formation of autophagosomes in DHAV-1-infected cells with rapamycin (RAPA)-treated cells and mock cells used as controls. Ultrastructural results showed that double-membraned autophagy-like vesicles formed in DHAV-1-infected cells and RAPA-treated cells but not in mock cells (Figure 8A). Notably, both the proteomic analysis and Western blot revealed that the expression levels of DAPK2 and sequestosome 1 (p62/SQSTM1) protein increased with DHAV-1 infection while their expression levels decreased with 4-phenylbutyrate (4-PBA) treatment (Figure 8B). We also found that the transformation from microtubule-associated protein 1 light chain 3-I (LC3I) to light chain 3-II (LC3II) was obviously increased in DHAV-1-infected cells (Figure 8C). These results revealed that autophagy could be initiated by DHAV-1, which might limit the degradation process.

With Hsps upregulated and ribosome proteins downregulated in our proteomic data, the expression level of the ER stress chaperone, glucose-regulated protein 78 (GRP78), was detected. Western blot results showed that the GRP78 protein was highly expressed in DHAV-1 infection, suggesting that DHAV-1 initiated the ER stress response (Figure 8D). To further verify the relationship between ER stress and autophagy that might exist in DHAV-1-infected cells, we examined the expression change in the transformation of LC3II/LC3I when cells were treated with 4-phenylbutyrate (4-PBA). The results showed that the transformation rate of LC3II/LC3I significantly increased compared with the mock cells, but it appeared to decrease in the 4-PBA-treated cells (Figure 8E).

## 3. Discussion

There is increasing application in proteomic analysis to identify the global changes of host proteins that are affected by viruses, stress, or abiotic factors [31,32,33]. Proteomic methods in virology can uncover antiviral responses, virus replication, and even the viral mechanisms of escape from host immune responses at the protein level. DEF cells have been used extensively to explore the molecular biological properties of DHAV-1 [34]. In this study, a quantitative proteomic analysis of DHAV-1-infected DEF cells at the whole cell protein level was performed using the TMT-labeling proteomic technique. We found 507 DEPs, among which 171 were significantly higher in abundance and 336 were in lower abundance in the D group. By referring to previous virus–host interaction analyses regarding the different virulence of DHAV-1 [22], immune-related genes, such as retinoic acid-inducible gene I (*RIG-1*) and melanoma differentiation associated gene 5 (*MDA5*), were significantly expressed in both studies. Common regulatory biological processes were also observed. For example, both studies revealed that host proteins were involved in “lysosome abundance”. The identification of these regulated proteins and processes in both studies confirmed the changes in lysosome abundance during DHAV-1 infection. Moreover, DEPs involved in ER stress and other metabolic processes were also found in our study. Proteomics analysis has previously showed changes and interactions in mitochondrial, ER, and ribosomal proteins of SH-SY5Y cells after oxygen and glucose deprivation [35]. In the present study, DEPs involved in ER stress and other metabolic processes were also identified. Further studies regarding these processes should be performed to increase our understanding of the pathogenic mechanism of DHAV-1.

It is essential in host defense that the cell maintains homeostatic processes regarding cell response to virus infection. By referring to GO enrichment, we found many biological processes and protein families that were significantly regulated and participated in cellular metabolism during DHAV-1 infection (Figure 5). It has been reported previously that the UPR regulator and ER chaperone, GRP78/BiP, was required for stress-induced autophagy [36]. A major proteomic response in the ER was identified after DHAV-1 infection. Activating transcription factor 3 (ATF3), which is involved in the external stimulus process, is integral to the double-stranded RNA-activated protein kinase (PKR)-like endoplasmic reticulum kinase (PERK)/eukaryotic initiation factor-2-α (eIF2α) signaling branch of the UPR [37]. In the present study, a major proteomic response in the ER was identified and ATF3 was upregulated in DHAV-1-infected DEF cells. Hsps, proteins that function in response to stimuli, and the ribosome protein family have been reported to be involved in cell stress, according to previous studies [38,39,40,41]. These proteins were identified as DEPs in the DHAV-1-infected DEF cells (Table 2), therefore they might be responsible for cell stress adaptation caused by DHAV-1 infection.

The acute decline of ribosome proteins is an implication that the host cell is deprived of a physiological equilibrium. The upregulation of GRP78 in DHAV-1-infected DEF cells (Figure 8) further supported the theory that ER stress was activated by DHAV-1. Ribosomal proteins, such as RPL31, RPL11, RPL38, RPL35, and RPL35A, were downregulated and formed a high interaction network (Figure 6), which may play roles in protein translation and ER docking, as per previous results [42]. Importantly, these ribosome proteins have relationships with immune-related proteins (red node in Figure 6), suggesting that the maintenance of cellular homeostasis requires complex co-regulatory interaction networks.

Autophagy, a key process in housekeeping and balance of the cell, can be initiated to maintain the synthesis, degradation, and subsequent recycling of cellular components when ER homeostasis cannot be rescued by the unfolded protein response (UPR) caused by a virus [43]. DAPK2 has been found to be a novel regulator of autophagy under stress and steady-state conditions by suppressing the mammalian target of rapamycin (mTOR) activity [44]. In the present study, both the proteomic data and Western blot assay results showed that the level of autophagy increased and DAPK2 was highly expressed in DHAV-1-infected cells. Previous studies showed that ER stress was an essential step for autophagy initiation, and pretreatment with 4-PBA decreased autophagy, illustrating that there was a close relationship between autophagy activation and ER stress [45,46]. However, whether the initiation of autophagy induced by DHAV-1 is related to ER stress is still unclear. In DHAV-1-infected cells, we observed the formation of autophagosomes, an increase in expression from LC3I to LC3II, and a decrease in expression levels of LC3 II protein when treated with 4-PBA (Figure 8). These results indicated that the DHAV-1 infection triggered the ER stress and autophagy processes, and ER stress was a key promoter in the induction of autophagy in the DHAV-1-infected DEF cells.

It was reported that the inhibition of autophagic flux may provide membranous surfaces for the assembly of viral RNA replication complexes and allow the extracellular release of viruses without cell lysis [47]. In theory, the formation of autophagosomes in DHAV-1-infected DEF cells could lead to a decrease in their degradation. However, in the present study, the less abundant proteins were significantly enriched in the lysosome and phagosome pathways, and p62 protein was more abundant, signifying that the fusion of autophagosomes with lysosomes might be limited in the DHAV-1 infection process. These results indicated that DHAV-1 may have adapted to evade autophagy to promote its infection and replication by preventing the degradation of autolysosomes, which was consistent with results regarding the coxsackievirus B3 infection [48].

## 4. Materials and Methods

### 4.1. Cells, Viruses, and Antibodies

DEF cells were obtained from duck embryos that were specifically pathogen-free for 10 days, as described previously [49]. DEF cells were cultured into Dulbecco’s modified eagle medium (DMEM) (Gibco, Carlsbad, CA, USA) with 10% fetal bovine serum (FBS, Gibco, USA) and antibiotics (100 IU/mL penicillin and 100 IU/mL streptomycin) at 37 °C under an atmosphere of 5% CO_2_/95% air.

DHAV-1 LY0801 strain (accession no. FJ436047) is a virulent strain, which was isolated in 2008 from an outbreak of severe duck virus hepatitis (DVH) in the Shandong province of China and kept in the veterinary molecular etiology laboratory of Shandong Agricultural University [2].

Anti-DHAV-1 monoclonal antibody (mAb) 4F8, whose epitope is “_75_GEILT_80_” in the VP1 protein of DHAV-1, was stored in our laboratory [50]. Fluorescein isothiocyanate (FITC)-labeled goat anti-mouse antibody was purchased from KPL (Gaithersburg, MD, USA). Other antibodies used in the Western blot assay in this study were purchased from Abcam (Cambridge, MA, USA).

### 4.2. Indirect Immunofluorescence Assay

DEF cells in 12-well plates were infected with DHAV-1. At 48 hpi, cells were washed with phosphate-buffered saline (PBS) and fixed with a mixture of acetone and formaldehyde (1:1, *v*/*v*) for 15 min at room temperature. Then, the cells were incubated with anti-DHAV-1 mAb 4F8 (dilution of 1:1000 with PBS) for 1 h at 37 °C. After being washed five times with PBS, FITC-conjugated goat anti-mouse IgG (Sigma, St. Louis, MO, USA) at a 1:200 dilution was added and incubated for 1 h at 37 °C in the dark. Finally, the plates were washed five times with PBS and observed using fluorescence microscopy (Leica AF6000, Leica, Wetzlar, Germany).

### 4.3. Quantitative of DHAV-1

Non-infected and DHAV-1-infected DEF cells were collected every 12 h from 12 to 72 hpi, and intracellular mRNA was extracted using Trizol reagent (Promega, Madison, WI, USA). The extracted RNA was then used for qRT-PCR quantification using the ABI 7500 (Applied Biosystems, Carlsbad, CA, USA) real-time PCR system according to the methods constructed by our lab [51]. Each experiment was replicated three times. The cycle threshold (Ct) value of each sample was used for data analysis.

### 4.4. Sample Preparation and TMT Labeling

DEF cells were infected with 2 MOI DHAV-1 for 2 h at 37 °C, then cells were washed with PBS and replaced with fresh DMEM with 2% FBS. At 48 hpi, cells of the D group or the N group were collected and centrifuged at 2000 rpm for 10 min to obtain the cell pellet. Each group was processed with three independent biological replications. These samples were sonicated three times on ice by using a high intensity ultrasonic processor (Scientz) in lysis buffer (8 M urea, 1% Protease Inhibitor Cocktail). After centrifugation at 12,000 g for 10 min, the remaining debris was removed, and the supernatant was collected. The concentration of proteins was determined with a bicinchoninic acid (BCA) kit (Beyotime Biotechnology, Shanghai, China), according to the manufacturer’s instructions. The extracted proteins were reduced with 5 mM dithiothreitol (DTT) at 56 °C for 30 min and alkylated with 11 mM iodoacetamide (IAA) in the darkness for 15 min at room temperature. After being diluted with 100 mM triethylammonium bicarbonate (TEAB), the proteins were digested with trypsin (Promega) at a ratio of 1:50 (trypsin/protein w/w). After trypsin digestion at 37 °C overnight, the peptide was desalted using a Strata X C18 SPE column (Phenomenex, Torrance, CA, USA) and vacuum-dried. The peptide was reconstituted in 0.5 M TEAB (Sigma, St. Louis, MI, USA) and processed according to the manufacturer’s protocol for the TMT kit (Thermo Fisher Scientific, Waltham, MA, USA). The peptide mixtures were then incubated for 2 h at room temperature, pooled, and desalted, and then dried using vacuum centrifugation.

### 4.5. HPLC Fractionation and LC-MS/MS Analysis

The labeled peptides were fractionated using high pH reverse-phase high pressure liquid chromatography (HPLC) with an Agilent 300Extend C18 column (5 μm particles, 4.6 mm ID, 250 mm length, Agilent, Santa Clara, USA). Briefly, peptides were first separated with a gradient of 8% to 32% acetonitrile (pH 9.0) over 60 min into 60 fractions, and the peptides were combined into 18 fractions and dried via vacuum centrifugation. Peptides were dissolved in 0.1% formic acid (solvent A, FA) and directly loaded onto a home-made reversed-phase analytical column (15-cm length, 75 μm i.d.). The gradient was comprised of an increase from 6% to 23% solvent B (0.1% FA in 98% acetonitrile) over 26 min, 23% to 35% in 8 min, and climbing to 80% in 4 min. Finally, a holding phase at 80% for 3 min was performed, all at a constant flow rate of 400 nL/min on an EASY-nLC 1000 ultraperformance liquid chromatography (UPLC) system (Thermo Fisher Scientific, Waltham, MA, USA).

The peptides were subjected to national system of innovation (NSI) sourcing, followed by MS/MS in Q Exactive^TM^ Plus (Thermo Fisher Scientific, Waltham, MA, USA), which was coupled online to the UPLC. Peptides were then selected for MS/MS using a normalized collisional energy (NCE) setting of 28 and the fragments were detected in the Orbitrap at a resolution of 17,500. A data-dependent procedure was performed, which involved alternating between one MS scan followed by 20 MS/MS scans with 15.0 s of dynamic exclusion. The automatic gain control (AGC) was set at 5E4 and the fixed first mass was set at 100 m/z. Three biological replicates were performed for each group.

### 4.6. Database Processing

The resulting MS/MS data were processed using the Maxquant search engine (v1.5.2.8). Tandem mass spectra were searched against the UniProt Anas platyrhynchos protein database (http://www.uniprot.org, 16377 sequences) and concatenated with a reverse decoy database. This target-decoy search strategy was employed to calculate the false-discovery rates (FDRs) [52]. The setting parameters of the proteome analysis were as follows: (1) The trypsin/P, trypsin; (2) 2 missing cleavages were allowed; (3) the minimum length of the peptide was 7; (4) the maximum number of modifications per peptide was 5; (5) the mass tolerance for precursor ions of the first search and the main search were 20 and 5 ppm, respectively; (6) the mass tolerance for fragment ions was 0.02 Da; (7) the fixed modification was Cys carbamidomethyl; and (8) the variable modification was Met oxidation. The FDR was adjusted to <1% and the minimum score for the peptides was set to >40.

### 4.7. Bioinformatics Analysis

GO annotation analysis was derived from the UniProt-GOA database (http://www.ebi.ac.uk/GOA/) [53]. The KEGG database was used to annotate the protein pathway and a two-tailed Fisher’s exact test was used to test the enrichment of the differentially expressed protein against all identified proteins [54]. The functional descriptions of the identified protein domains were annotated using InterProScan (a sequence analysis application) based on the protein sequence alignment method, and the InterPro domain database was used [55]. GO, the KEGG pathway, and protein domain enrichment were all performed with a corrected *P*-value of <0.05. This filtered *P*-value matrix was transformed via the function x = −log10 (*P*-value). Finally, these x values were z-transformed for each functional category and the z scores were clustered by one-way hierarchical clustering (Euclidean distance, average linkage clustering) in Genesis. Cluster membership was visualized with a heat map using the “heatmap.2” function from the “gplots” R-package. All DEP sequences were searched against the STRING database version 10.5 for protein–protein interactions (PPIs). All interactions that had a confidence score ≥0.7 (high confidence) in the STRING database were retrieved for the analysis. The interaction network obtained from STRING was visualized in Cytoscape software (version 3.0.1) [56].

### 4.8. PRM Analysis

The peptide samples were prepared according to the whole cell proteome analysis methodology described above. The proteins selected for PRM validation were based on the proteome results. The peptide sample was initially run in a data-dependent acquisition (DDA) mode using Q Exactive^TM^ Plus (Thermo Fisher Scientific, Waltham, MA, USA), which was coupled to the UPLC online with an identical gradient to subsequent PRM analysis. The electrospray voltage applied was 2.0 kV. A full mass spectrum was detected using the Orbitrap at a resolution of 35,000 (the m/z scan range was 350 to 1000), followed by 20 MS/MS scans using the Orbitrap at a resolution of 17,500 (the automatic gain control (AGC) target was 3E^6^ and the maximum injection time was 20 ms) in a data-independent procedure. The isolation window for MS/MS was set at 2.0 m/z. The NCE was 27% with higher energy collisional dissociation (HCD). Three biological replicates were performed.

### 4.9. Transmission Electron Microscopy

DEF cells were infected with DHAV-1 (MOI = 2) and maintained in 2% DMEM. At 48 hpi, cells were processed for EM according to standard procedure. Simply, cells were fixed with fixing solution for 2 h at 4 °C. The cell pellet was embedded with 1% agarose and washed three times with 0.1 M PBS (pH 7.4). Cells were post-fixed with 1% OsO_4_ in 0.1 M PBS (pH 7.4) at room temperature for 2 h. After rinsing in 0.1 M PBS, samples were processed via a series of dehydration steps ranging from 50% to 100% ethanol, with a final 100% acetone incubation. Samples were infiltrated with 1:1 acetone:EMBed 812 for 2 to 4 h, then with 2:1 acetone:EMBed812 overnight, and finally with pure EMBed 812 for 5 to 8 h. The resin was polymerized with embedded cells at 60 °C for 48 h. Sections measuring 80 nm were cut with ultramicrotome and stained with uranyl acetate in pure ethanol for 15 min, then rinsed with distilled water. Finally, images were observed with TEM.

### 4.10. Western Blotting

DEF cells were infected with DHAV-1 (2 MOI) as described above, and cell proteins were extracted at the indicated time. DEF cells were treated with 2.5 μg/mL tunicamycin (Tm), 6 μM RAPA, or 4ug/mL 4-PBA for 24 h. Extracted protein mixtures were separated using sodium dodecyl sulfate-polyacrylamide gel electrophoresis (SDS-PAGE) gel and transferred to poly-vinylidene difluoride (PVDF) membranes using the Trans-Blot Turbo Transfer System (BIO-RAD Laboratories, Berkeley, CA, USA), which was then blocked with 5% nonfat milk. The membranes were incubated with a specific primary antibody diluted in primary antibody dilution buffer (Beyotime Biotechnology, Shanghai, China) overnight at 4 °C. After washing with tris-buffered saline with 0.1% Tween 20 (TBST), the membranes were incubated with specific secondary antibodies for an additional 2 h at room temperature. Finally, the immune complexes were visualized with an enhanced chemiluminescence (ECL) detection kit (Beyotime Biotechnology, Shanghai, China), and protein expression levels were analyzed using Image J software (Rawak Software, Inc., Munich, Germany).

## 5. Conclusions

In conclusion, using proteomic technology, a total of 507 DEPs were identified in DHAV-1-infected DEF cells. The proteomic data, TEM, and Western blot results indicated that DHAV-1 infection triggered ER stress and the autophagy process, and that ER stress was a key promoter in the induction of autophagy in DHAV-1-induced DEF cells. To the best of our knowledge, this was the first report regarding the utilization of proteomic technology to investigate DHAV-1–host interactions, and these findings could pave a new way toward uncovering the regulatory mechanisms behind DHAV-1 infection.

## Figures and Tables

**Table 1:** Summary of MS/MS spectra database search analysis.

Total Spectra	Matched Spectra	Peptides	Unique Peptides	Identified Proteins	Quantifiable Proteins
273,692	47,151 (17.2%)	29,975	28,990	5250	4573

**Table 2:** A partial result of the differentially expressed proteins of the DHAV-1-infected group (D) compared to the non-infected group (N).

Protein ID	Description	D/N Ratio	*P*-Value
**Heat Shock Proteins**			
R0K012	Activator of 90 kDa heat shock protein ATPase-like protein 2	1.35	0.0086
U3IGK2	DnaJ heat shock protein family (Hsp40) member C21	1.355	0.00424
U3IPI8	Heat shock protein family B (small) member 8	1.773	0.0000227
U3IZQ3	Heat shock protein family B (small) member 7	2.034	0.000119
**Ribosome Related Proteins**			
U3IZF8	Ribosomal protein L11	0.691	0.000158
U3IPM7	Ribosomal protein L38	0.732	0.000925
U3J6F1	Ribosomal protein L35a	0.751	0.000819
R0L8K3	60S ribosomal protein L35	0.643	0.000863
U3JA05	Ribosomal protein L31	0.74	0.0000179
U3IU44	60S ribosomal protein L36	0.516	0.000141
U3IA42	Mitochondrial ribosomal protein L27	0.765	0.0119
U3IK48	Ribosomal protein S5	0.658	0.000638
U3J834	Ribosomal protein S19	0.757	0.000105
**Zinc Finger Proteins**			
U3IIQ5	Zinc finger E-box binding homeobox 1	1.345	0.0414
U3IUP4	NFX1-type zinc finger-containing protein 1	2.675	0.0000234
**Response to Stimulus**			
U3IWA4	Apolipoprotein B	1.388	0.00256
U3ITV8	Interferon induced with helicase C domain 1	1.986	0.0000433
U3I515	eukaryotic translation initiation factor 2 alpha kinase 2	3.417	0.00000138
U3IQI3	F-box protein 18	2.468	0.0000779
S4SM19	ATP-dependent RNA helicase	2.806	0.0000825
U3J4N1	SAM and HD domain containing deoxynucleoside triphosphate triphosphohydrolase 1	1.828	0.0000226
U3IQ81	Adenosine deaminase, RNA specific	1.67	0.0000588
U3J485	ATP binding cassette subfamily A member 1	1.414	0.001
U3IRD3	Ankyrin repeat domain 1	1.567	0.00122
U3I3I3	Beta-2-microglobulin	2.718	0.0000783
U3IED8	Radical S-adenosyl methionine domain containing 2	4.454	0.0000988
R0LWV1	Activating transcription factor 3	1.737	0.000138
**Immune-Related Proteins**			
U3IXS5	Tripartite motif containing 35	1.5	0.0000634
U3I1L8	Tripartite motif containing 59	1.38	0.000201
U3J0V8	Signal transducer and activator of transcription 1	2.985	0.0000419
U3IB66	Tripartite motif containing 25	3.469	0.0000000192
U3I8M2	Interferon induced protein 35	2.716	0.000245
U3ITV8	Interferon induced with helicase C domain 1	1.986	0.0000433
U3I515	eukaryotic translation initiation factor 2 alpha kinase 2	3.417	0.00000138
U3IRN3	S100 calcium binding protein A12	2.687	0.0000754
U3IGN0	N-myc and STAT interactor	1.408	0.000302
U3J6Y2	ISG15 ubiquitin-like modifier	9.858	0.00000348
U3I1B1	IFN-induced protein with tetratricopeptide repeats 5	8.506	0.00104
**Autophagy-Associated Proteins**			
U3I5T9	V-type proton ATPase subunit	0.493	0.0000955
U3IIS7	Integrin beta	0.583	0.00000173
U3IN31	N-acetylglucosamine-6-sulfatase	0.732	0.000137
U3IYU5	Cathepsin S	0.63	0.000337
U3I5T9	V-type proton ATPase subunit D	0.493	0.0000955
U3I832	V-type proton ATPase subunit H	0.769	0.00176
U3IST9	V-type proton ATPase subunit C	0.541	0.00000192
U3IMC2	Sequestosome 1/SQSTM1	1.336	0.000459
U3I4S4	Death associated protein kinase 2	1.374	0.000564

## Supplementary Materials

Supplementary materials can be found at https://www.mdpi.com/1422-0067/20/24/6160/s1. Table S1. List of proteins identified and quantified by the TMT analysis in the experiment. Table S2. List of differentially expressed proteins. Table S3. GO annotation. Table S4. Subcellular location. Table S5. Enrichment analysis of DEPs based on GO annotation in upregulated proteins. Table S6. Enrichment analysis of DEPs based on GO annotation in downregulated proteins. Table S7. Enrichment analysis of the DEPs according to KEGG pathway (upregulated). Table S8. Enrichment analysis of the DEPs according to KEGG pathway (downregulated). Table S9. Enrichment of the DEPs according to domain (upregulated). Table S10. Enrichment of the DEPs according to domain (downregulated). Table S11. Interaction networks of DEPs.

## Abbreviations

4-PBA	4-phenylbutyrate
ADAM17	ADAM metallopeptidase domain 17
AGC	Automatic gain control
AGC	Automatic gain control
ATF3	Activating transcription factor 3
BCA	Bicinchoninic acid
DAPK2	Death-associated protein kinase 2
DDA	Data-dependent acquisition
DEF	Duck embryo fibroblast
DEPs	Differentially expressed proteins
DHAV-1	Duck hepatitis A virus type 1
DMEM	Dulbecco’s modified eagle medium
DTT	Dithiothreitol
DVH	Duck virus hepatitis
ECL	Enhanced chemiluminescence
ER	Endoplasmic reticulum
FA	Formic acid
FBLN2	Fibulin 2
FBS	Fetal bovine serum
FDR	False discovery rates
FITC	Fluorescein isothiocyanate
GO	Gene Ontology
GRP78	Glucose-regulated protein 78
HCD	Higher energy collisional dissociation
Hpi	Hours post-infection
HPC	High pressure liquid chromatography
Hsps	Heat shock proteins
IAA	Iodoacetamide
IFIH1	Interferon-induced helicase
IFIT5	Interferon-induced protein with tetratricopeptide repeats 5
ISG15	Interferon-stimulated gene 15
ITGB2	Integrin beta 2
KEGG	Kyoto Encyclopedia of Genes and Genomes
LC3-I	Microtubule-associated protein 1 light chain 3-I
LC3-II	Microtubule-associated protein 1 light chain 3-II
LCP1	Lymphocyte cytosolic protein 1
mAb	Monoclonal antibody
MDA5	Melanoma differentiation associated gene 5
MOI	Multiplicity of infection
MOV10	Mov10 RISC complex RNA helicase
MS/MS	Tandem mass spectrometry
Mx1	Myxovirus resistance gene 1
MYL1	Myosin light chain 1
NCE	Normalized collisional energy
NSI	National system of innovation
p62/SQSTM1	Sequestosome 1
PARP9	Poly(ADP-ribose) polymerase 9
PBS	Phosphate-buffered saline
PERK	Double stranded RNA activated protein kinase (PKR)-like endoplasmic reticulum kinase
PPI	Protein-protein interactions
PRM	Parallel reaction monitoring
PVDF	Poly-vinylidene difluoride
RAPA	Rapamycin
RIG-1	Retinoic acid-inducible gene 1
RSAD2	Radical S-adenosyl methionine domain containing 2
SDS-PAGE	Sodium dodecyl sulfate-polyacrylamide gel electrophoresis
TBST	Tris-buffered saline with 0.1% Tween 20
TEAB	Triethylammonium bicarbonate
TEM	Transmission electron microscopy
TMT	Tandem Mass Tag
TNC	Tenascin C
TRIM25	Tripartite motif containing 25
UPLC	Ultraperformance liquid chromatography
UPR	Unfolded protein response
ZFP	Zinc finger proteins
